# Stability and Synergistic Effect of Polyaniline/TiO_2_ Photocatalysts in Degradation of Azo Dye in Wastewater

**DOI:** 10.3390/nano7120412

**Published:** 2017-11-23

**Authors:** Vanja Gilja, Katarina Novaković, Jadranka Travas-Sejdic, Zlata Hrnjak-Murgić, Marijana Kraljić Roković, Mark Žic

**Affiliations:** 1 Faculty of Chemical Engineering and Technology, University of Zagreb, Marulićev trg 19, 10000 Zagreb, Croatia; vgilja@fkit.hr (V.G.); kata.novakovic@gmail.com (K.N.); zhrnjak@fkit.hr (Z.H.-M.); mkralj@fkit.hr (M.K.R.); 2 School of Chemical Sciences, University of Auckland, Polymer Electronics Research Centre, 23 Symonds Street, Auckland 1010, New Zealand; j.travas-sejdic@auckland.ac.nz; 3 Ruđer Bošković Institute, Bijenička cesta 54, 10000 Zagreb, Croatia

**Keywords:** wastewater treatment, Reactive Red 45, photocatalysis, UVA/Vis irradiation, polyaniline/TiO_2_, composite photocatalysts, in situ synthesis

## Abstract

The polyaniline/TiO_2_ (PANI/TiO_2_) composite photocatalysts were prepared by the in situ chemical oxidation of aniline (An) in the presence of TiO_2_ particles. For this purpose, photocatalysts with different amounts of PANI polymer were prepared and analysed. Fourier-transform infrared (FT-IR) spectroscopy and thermogravimetric (TG) analysis indicated successful synthesis of the PANI polymer and its conductivity was also determined. The micrographs of field emission scanning electron microscopy (FE-SEM) and transmission electron microscopy (TEM) were used to explain the impact of the aniline amount on the aggregation process during the synthesis of the composites. The smallest size of aggregates was obtained for the photocatalysts with 15% of PANI (15PANI/TiO_2_) due to the formation of homogenous PANI. The photocatalytic activity of studied PANI/TiO_2_ photocatalysts was validated by monitoring the discoloration and mineralization of Reactive Red azo dye (RR45) in wastewater. The 15PANI/TiO_2_ sample presented the highest photocatalytic efficiency under ultraviolet A (UVA) irradiation, in comparison to pure TiO_2_. This was explained by the formation of uniformly dispersed PANI on the TiO_2_ particles, which was responsible for the synergistic PANI-TiO_2_ effect.

## 1. Introduction

Organic synthetic dyes (e.g., Reactive Red (RR45)), originating from textile and dye industries, are among prominent environmental pollution factors. To dismiss the possibility of pollution, organic dyes have been removed from wastewaters, both by discoloration and complete mineralization [1]. One of the most efficient approaches to degrading organic dye is to apply advanced oxidation processes (AOPs), with the aim of converting dyes to non-toxic and non-hazardous species that can be released in the environment [2].

Photocatalysis is a widely studied process, which utilizes light to activate semiconductor metal oxide catalysts. Furthermore, titanium dioxide (TiO_2_) is one of the most commonly used commercial photocatalysts, as it is non-toxic, low in cost and environment friendly, due to its chemical stability [3,4,5]. However, the relatively high band gap (3.2 eV) of TiO_2_ [6] restricts its photocatalytic activity under visible light (Vis), whereas its poor quantum efficiency, due to recombination of electrons (e_CB_^−^) and holes (h_VB_^+^) [7], is another disadvantage. 

However, TiO_2_ photocatalytic activity under Vis (>400 nm) can be increased by doping [6]; and thus, numerous groups utilize transition metal ions, such as Fe, Au, Ag, V, Cr and Ni, to hinder the recombination process [1,8,9,10]. The recombination process in TiO_2_ has also been suppressed by preparing polyaniline/TiO_2_ (PANI/TiO_2_) composites [11], which is a promising, though poorly investigated approach. The advantage is that PANI is a conductive, non-toxic and thermally stable polymer, with a high absorption coefficient in the Vis spectrum [12].

PANI is capable both of injecting e_CB_^−^ (obtained by absorption of a photon from Vis irradiation) into the TiO_2_ conduction band (CB), and accepting h_VB_^+^ from the TiO_2_ valence band (VB) [13]. Thus, the aforementioned PANI-TiO_2_ interactions result in the PANI-TiO_2_ synergistic effect that hinders the recombination process and increases TiO_2_ activity under ultraviolet (UV) and Vis irradiation [13]. Photocatalysis then proceed by the typical mechanism, known for TiO_2_: e_CB_^−^ and h_VB_^+^ transform H_2_O and O_2_ into OH radicals, which in turn degrade the organic pollutants [14,15].

PANI/TiO_2_ composites can be prepared by in situ chemical oxidation of aniline in the presence of TiO_2_ particles. Generally, aniline oxidation can yield different products as oligomers and oxidation products with an amino group in the *ortho*-position [16], which presents imperfections that might influence aggregate size. In addition, PANI properties were shown to be perturbed when monomers with amino group in the *ortho*-position were added to the synthesis solution [17,18,19]. Thus, the optimal PANI-TiO_2_ synergistic effect can be obtained only if the synthesis conditions are carefully chosen. However, in literature to date, the impact of the initial synthesis conditions on the in situ PANI/TiO_2_ synthesis has not been clarified yet.

In the present study, PANI/TiO_2_ composites were prepared by chemical in situ oxidation of aniline, in the presence of TiO_2_, by using ammonium persulfate (APS) as the oxidant [13]. In order to obtain the PANI-TiO_2_ synergistic effect, the polymer has to be in its conductive form [12] and the aggregates should be of optimal size. However, the PANI conductivity and aggregate sizes are properties that are governed by the synthesis conditions: the concentrations of An and APS, the An:APS ratio, the type of acid, pH, etc. [16]. 

The objective of this study was to synthesize PANI/TiO_2_ composite photocatalysts, (i) to study stability as well as the composition and structure of PANI that are essential for the synergistic PANI-TiO_2_ effect; (ii) to prepare PANI/TiO_2_ composite photocatalysts with higher photocatalytic efficiencies under UVA irradiation, in comparison to pure TiO_2_ and (iii) to explain the impact of aniline concentration on the in situ PANI/TiO_2_ composite synthesis. Special attention was focused on explaining the impact of aniline concentration on the aggregation process and on the PANI-TiO_2_ synergistic effect.

## 2. Materials and Methods

### 2.1. Materials

TiO_2_ (P-25, Aeroxide) from Evonik (Degussa, Essen, Germany), aniline (An, 99%), ammonium persulfate (APS) and H_2_SO_4_ (96%) from Acros Organics (Morris Plains, NJ, USA) were used to synthesize samples. The chemicals were used as received, without any additional pre-treatment.

### 2.2. PANI/TiO_2_ Photocatalyst Preparation

The aniline monomer was oxidized by APS in the presence of TiO_2_ particles. In situ polymerization processes were conducted at a constant mole ratio of n(An):n(APS) = 1:0.25. Aniline additions were predetermined to yield composites in which the weight ratio of PANI in the composites were 10%, 15%, 20% and 25% (Table 1). A quantity of 0.8 g TiO_2_ was used in all preparation procedures. 

In the case of the 10PANI/TiO_2_ composite, the weight ratio of the oxidized aniline (PANI) vs. TiO_2_ was 0.10. Also, other photocatalysts were synthesized in the same manner as 10PANI/TiO_2_ sample, as follows: (a) 50 mL aqueous A solution was prepared using 0.8 g TiO_2_ and 0.055 mL H_2_SO_4_ and sonicated for 15 min to obtain a stable suspension; (b) 50 mL aqueous B solution was prepared using 0.392 mL An and 0.055 mL H_2_SO_4_; and (c) 50 mL aqueous C solution was prepared using 0.245 g APS and 0.055 mL H_2_SO_4_ (Table 1). In order to obtain a stable An–TiO_2_ suspension, the A and B solutions were mixed and stirred (500 rpm) in a reactor container for a period of 15 min. Next, the in situ polymerization process was commenced by adding C solution to the reactor, which was refilled with H_2_O, to obtain the total reaction volume of 200 mL. The reactor suspension was stirred for 24 h at room temperature. The pure PANI sample was prepared by the same procedure used to prepare 10PANI/TiO_2_, but without adding TiO_2_. The in situ polymerization process resulted in a dark green product that was washed only by using H_2_O and centrifugation and afterwards dried at 60 °C for 24 h. 

### 2.3. Characterizations

FT-IR spectra were obtained using a Spectrum One FT-IR spectrometer (Perkin Elmer, Shelton, CT, USA) in the range of 4000–650 cm^−1^. UV/Vis spectra were made over the range, 200 to 800 nm, with a spectral resolution of ~0.3–10 nm, using a UV/Vis spectrometer (Ocean Optics USB 2000+, Ocean Optics, Largo, FL, USA). SEM images were obtained by a thermal field emission scanning microscope (FE-SEM, model JSM-7000F, JEOL Ltd., Akishima, Japan). TEM images were obtained by a transmission electron microscope (FEI Tecnai FEG20, Tecnai, Hillsboro, OR, USA). The conductivity of PANI/TiO_2_ composites was measured by the four-point probe method (Keysigiht 34461 61/2 Digit Multimeter, Keysight Technologies, Santa Rosa, CA, USA). Thermogravimetric (TG) analyses of samples were performed using a TA Instruments Q500 analyser (TA Instruments, New Castle, DE, USA). The TG results were obtained in the temperature range from 25 °C to 800 °C, at a heating rate of 10 °C/min, under a nitrogen atmosphere.

### 2.4. Photocatalytic Activity Test

To investigate the PANI-TiO_2_ synergistic effect under UVA/solar irradiation, wastewater was prepared from the dispersion of 30 mg/L azo dye (Reactive Red 45) and 1 g/L photocatalyst. The solution was poured into a glass water-jacketed batch reactor equipped with both a Pen-Ray UVP lamp, emitting irradiation in the UVA region (315 to 400 nm) and a solar irradiation simulator Oriel Newport (Osram XBO 450 W lamp, Oriel Instruments, Irvine, CA, USA). The total volume of stirred (250 rpm) solution during the experiments was 75 mL. Prior to performing photocatalysis, the adsorption equilibrium was reached by leaving the samples in the dark for 30 min; and consequently, the photocatalysis process was carried out for another 90 min. Decolorization of RR45 in solution was monitored by a Perkin Elmer Lambda EZ 201 UV/Vis spectrophotometer (Perkin Elmer, Shelton, CT, USA), where the absorption was measured at a wavelength of 542 nm for RR45. Before the decolorization measurement, samples were taken from the reactor (every 15 min) and filtered using Chromafil XTRA RC (25 mm, 0.45 micron, Macherey-Nagel, Düren, Germany) filters. The results were presented as removed dye from wastewater after treatment, and they were determined by calculation, according to the equation:

$$\frac{A_{t}}{A_{0}}$$

where A_0_ and A_t_ are adsorption values at t = 0 and t = t.

The extent of mineralization of RR45 dye was determined as the concentration of total organic carbon (TOC) after photocatalysis, using the Total Organic Analyser: Shimadzu TOC-VCPN (Shimadzu, Kyoto, Japan).

## 3. Results and Discussion

### 3.1. FT-IR Analyses of Photocatalysts

**Figure 1:**
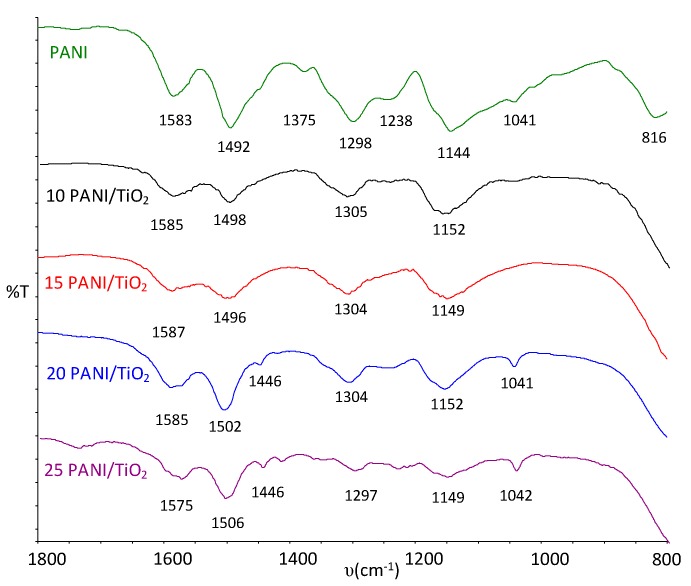
FT-IR spectra of synthesized PANI and different PANI/TiO_2_ composite photocatalysts.

The PANI-TiO_2_ synergistic effect is obtained when PANI is in the conductive form [13]. Thus, to collect data relevant for PANI conductivity and PANI-TiO_2_ interactions, the samples were examined using FT-IR spectroscopy. Figure 1 shows that pure PANI is characterized by vibrations at 1298 cm^−1^ and 1238 cm^−1^ that are attributed to C-N stretching modes, whereas C-H out of plane vibrations are positioned at 1144 cm^−1^ and 816 cm^−1^ [20]. 

**Scheme 1:**
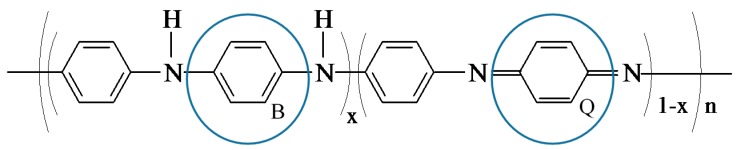
Structural formula of PANI and quinoid (Q) and benzoid (B) units.

All studied samples showed distinct bands in the ranges, 1575–1587 cm^−1^ and 1492–1502 cm^−1^, due to C=N and C=C stretching modes, typical for quinoid (Q) and benzoid (B) units (Scheme 1). The intensity ratio of bands typical for C=N and C=C stretching modes implied that PANI was in its conductive form [21,22]. 

**Scheme 2:**
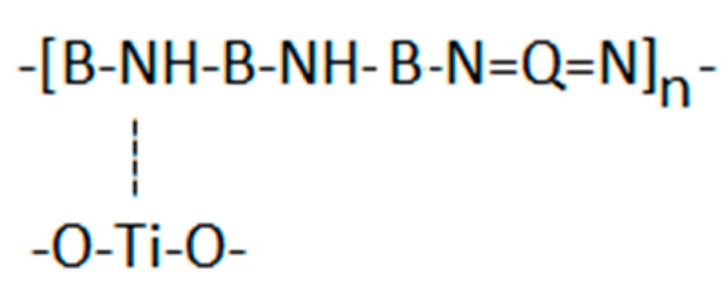
Established interactions in PANI/TiO_2_ composite between the nitrogen (N) in PANI and titanium (Ti) in TiO_2_.

Figure 1 shows the presence of the TiO_2_ particles that decreased transmission values at wavenumbers lower than 900 cm^−1^. However, vibration modes at 3630–3680 cm^−1^ and 3500–3420 cm^−1^, typical for stretching and bending vibrations of –OH groups at the TiO_2_ surface [23], were not observed in this work (not presented). Furthermore, Figure 1 indicates that the characteristic PANI peaks of C=N, C=C and C–N (1583 cm^−1^, 1492 cm^−1^, 1298 cm^−1^) in the 15PANI/TiO_2_ composite were shifted to higher wavenumbers (1587 cm^−1^, 1496 cm^−1^ and 1304 cm^−1^). Titanium, as a transition metal, has a tendency to form a coordination compound with nitrogen atoms (Ti–N) which could weaken the strength of the C=N, C=C and C–N bands, that shift typical PANI bands to higher wavenumbers [24]. Thus, the shift of the characteristic PANI peaks in 15PANI/TiO_2_ indicates the presence of PANI-TiO_2_ interactions that are obtained through Ti–N interactions (Scheme 2).

### 3.2. TG Analyses of Photocatalysts

**Figure 2:**
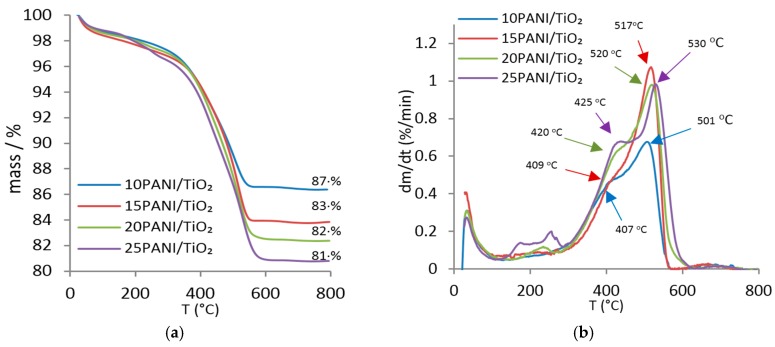
(**a**) Thermogravimetric (TG) and (**b**) dTG curves of the synthesized 10-25PANI/TiO_2_ composite photocatalysts.

In order to inspect the amount and stability of PANI in composites, the thermal decomposition of PANI/TiO_2_ photocatalysts was investigated by TG in a nitrogen atmosphere (Figure 2). The TG curves in Figure 2a suggest that the PANI mass loss occurred in one step, from 30 °C to 600 °C. Furthermore, it can also be seen that the char residue, containing mainly TiO_2_ particles, declined with increasing PANI amount. However, a more detailed inspection of the dTG curves in Figure 2b suggest that decomposition processes occurred in three different temperature stages.

The first stage, which was similar for all samples, can be attributed to water loss up to 160 °C. The second stage, from 160 °C to 400 °C shows the removal of dopant and imperfections (i.e., oligomers and products with amino groups in the *ortho*-position) [25]. Finally, the third stage (from 400 °C) is characterized by thermal decomposition (i.e., stability) of the main PANI chains [26]. It is well-known that conductive PANI begins to decompose at 370 °C under air conditions [27]. However, in nitrogen (vs. air) atmosphere, the PANI chain structure decomposes at higher temperatures (>400 °C) which indicates greater thermal stability (Figure 2b) [13,25,28]. The dTG curves show that the 25PANI/TiO_2_ sample, with the highest shoulder, experienced the most significant mass loss in the second stage, due to the highest concentration of dopants and imperfections (Figure 2b). 

Samples 10PANI/TiO_2_ and 20PANI/TiO_2_ presented shoulders in the second step and maxima positioned at 407 °C and 420 °C, respectively. On the other hand, the 15PANI/TiO_2_ composite was characterized by a single strong peak at 517 °C and by a small shoulder at 409 °C. It can be concluded that PANI chains of lower molar mass (and lower stability) decomposed in the second stage, whereas the third stage was characterised by disintegration of polymer chains of higher molar mass with greater temperature stability.

Furthermore, it is assumed that the concentration and chemical structure of PANI on TiO_2_ particles are the main factors impacting the PANI degradation rate. Figure 2b shows that 10PANI/TiO_2_ presented the lowest degradation rate (0.652%/min), while the highest degradation rate (1.075%/min) was observed for the 15PANI/TiO_2_ composite. However, when the PANI ratio was further increased, the 20–25PANI/TiO_2_ decomposition rate was reduced (≈0.98%/min). The decrease from 1.075 to ≈0.98%/min can be explained by the increase in PANI imperfection amount, which occurred due to the application of a higher aniline concentration. On the other hand, the low aniline concentration used to prepare 15PANI/TiO_2_, reduced PANI imperfections, which resulted in the highest composite decomposition rate.

### 3.3. Conductivity of Photocatalysts

The PANI/TiO_2_ composite conductivity varied with PANI concentration, according to the results shown in Table 2. The results indicate a slight variation in the 10–20PANI/TiO_2_ composite conductivity, whereas 25PANI/TiO_2_ had the lowest conductivity (1.70 × 10^−6^ S cm^−1^). According to the TG results, the lowest conductivity of the 25PANI/TiO_2_ composite was expected, due to the highest concentration of imperfections (Figure 2b). The lower conductivity may indicate that the main polymer chain of 25PANI/TiO_2_ is of a low degree of order. This arises from the fact that as the PANI chain is more linear, the diffusion of electrons along the chain is promoted, which consequently increases the DC conductivity [29,30,31]. 

In addition, the 10PANI/TiO_2_ sample had the lowest PANI amount, which consequently resulted in the lowest conductivity. It is evident for 10–20PANI/TiO_2_ samples, that the concentration of aniline monomers during in situ synthesis significantly affected the PANI amount in composites (Figure 2). Thus, an increase in composite conductivity (Table 2) occurred due to a higher amount of PANI in the composites.

### 3.4. UV-Vis Spectra of the Photocatalysts

**Figure 3:**
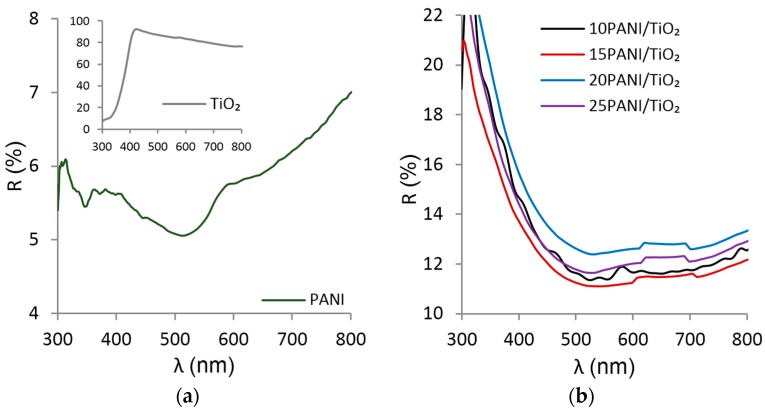
UV/Vis reflectance spectra of (**a**) the synthesized PANI and (**b**) PANI/TiO_2_ composite photocatalysts. The inset in the diagram (**a**) represents the UV/Vis reflectance spectrum of pure TiO_2_.

UV-Vis spectroscopy was used to characterize the synthesized PANI/TiO_2_ composite photocatalysts (Figure 3). The inset in Figure 3a indicates that pure TiO_2_ reflected >80% of the light in the Vis region (400–800 nm) in contrast to the pure PANI that reflected ≈6% (Figure 3a). However, the reflectance values of the PANI/TiO_2_ composites varied between those two extremes (i.e., pure PANI and pure TiO_2_). As PANI/TiO_2_ composites reflected a lower percentage of light than pure TiO_2_, it can be concluded that PANI was immobilized at the TiO_2_ surface.

Furthermore, the lowest intensity of reflected irradiation (≈11%) was observed for 15PANI/TiO_2_, which indicates that PANI homogenously covered the TiO_2_ surface. On the other hand, increased reflectance values for 20–25PANI/TiO_2_ (vs. 15PANI/TiO_2_) indicate a non-homogenous morphology, which was obtained due to a higher portion of imperfections, as verified by the TG analyses (Figure 2). Thus, the experimental conditions for 15PANI/TiO_2_ synthesis were optimal for yielding the homogeneous composite morphology.

### 3.5. FE-SEM and TEM Analyses

**Figure 4:**
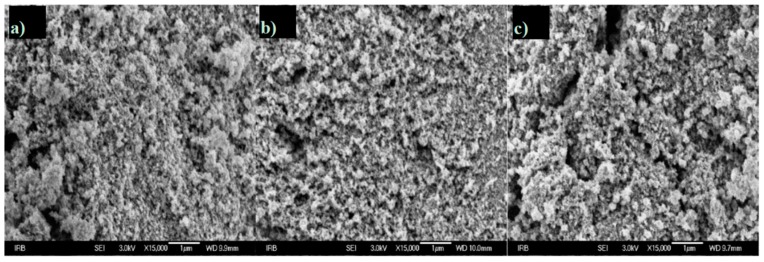
SEM micrographs of (**a**) 10PANI/TiO_2_; (**b**) 15PANI/TiO_2_ and (**c**) 20PANI/TiO_2_ composites (magnification ×15,000).

**Figure 5:**
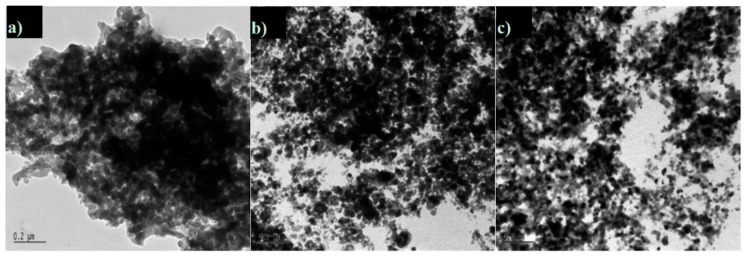
TEM image of (**a**) 10PANI/TiO_2_; (**b**) 15PANI/TiO_2_ and (**c**) 20PANI/TiO_2_ composite (magnification ×15.000).

The morphology of the 10–20PANI/TiO_2_ composites was analysed by FE-SEM and TEM and the micrographs are shown in Figure 4 and Figure 5. The SEM micrographs demonstrate composite aggregates of an average size of 200–1000 nm (Figure 4a–c), while the smallest ones (≈200 nm) are visible in the 15PANI/TiO_2_ composite (Figure 4b). Interestingly, the pure PANI agglomerates were not detected in Figure 4b, which agrees with conclusions that PANI in 15PANI/TiO_2_ mainly covers the TiO_2_ surface (see Section 3.4). Furthermore, the TEM images show that the aggregates are comprised of TiO_2_ particles with an average size of 10–20 nm (Figure 5a–c). The aggregate composition presented in Figure 5a–c corroborates previous literature reports [32], in which authors showed that the small (10–20 nm) TiO_2_ particles form large PANI/TiO_2_ aggregates. 

**Scheme 3:**
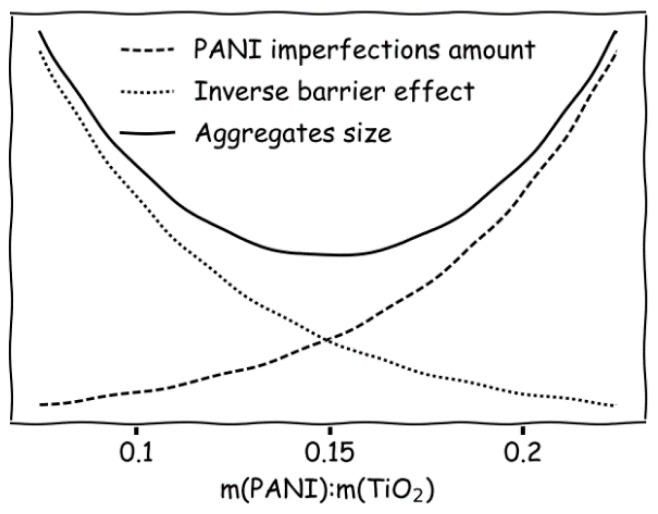
Schematic representation of the aniline amount impact on the PANI/TiO_2_ aggregate size, obtained by in situ aniline oxidation in the presence of TiO_2_ particles. The aggregate size minimum, which resulted in the most homogeneous morphology, was obtained for the 15PANI/TiO_2_ composite.

The PANI/TiO_2_ photocatalytic activity can be severely decreased if large aggregates are formed, due to TiO_2_ particle collision. In this study, frequent TiO_2_ particle collisions were expected, as a rather high TiO_2_ concentration (4 g/L) was used. Figure 5a shows a large aggregate (≈1000 nm), which confirms that the collision of TiO_2_ particles occurred and contributed to the formation of large composite particles. However, the literature to date does not offer any explanation of how aniline concentration influences the PANI/TiO_2_ composite aggregation process. Therefore, the following discussion and Scheme 3 provide an insight into the impact of aniline concentration on the aggregation process.

It is well-known that organic (e.g., aniline) molecules create a barrier (i.e., barrier effect) to the aggregation process of TiO_2_ nanoparticles [33]. Thus, a low aniline concentration facilitates the formation of large aggregates (Figure 5a). As expected, when the aniline concentration was augmented, the aggregate size was reduced to ≈200 nm (Figure 4b). Therefore, it is evident that an increased aniline amount hindered the TiO_2_ aggregation process. Furthermore, to facilitate the analyses in this work, the barrier effect was presented in the form of the “inverse” barrier effect (Scheme 3). 

Scheme 3 shows that an increase in aniline concentration yields smaller aggregate sizes, due to a lower inverse barrier effect. Unexpectedly, Figure 4c indicates that a further increase in monomer concentrations resulted in a larger aggregates size (≈350 nm). Furthermore, Figure 4 and Figure 5 suggest that the aggregate size is not governed only by the barrier effect. Thus, it can be concluded that the different synthesis products (i.e., imperfections) have an impact on the aggregate size, as presented in Scheme 3.

In acidic media (pH < 2.5), the aniline oxidation (without the presence of TiO_2_ particles) can produce (i) granules; (ii) layers; (iii) oligomers; (iv) dispersion of pure polymer in solution or (v) products with amino groups in the *ortho*-position [16]. In this study, due to a higher aniline concentration, a high portion of imperfections (i.e., oligomers and products with amino groups in the *ortho*-position [16]), was obtained in the 20–25PANI/TiO_2_ composites. For example, the presence of monomers with amino groups in the *ortho*-position in a synthesis solution can facilitate PANI chain branching [19,31]. Thus, branching through *ortho*-positions might facilitate connections between small composite particles in 20PANI/TiO_2_ (Figure 4c). Consequently, both the low aniline concentration in 10PANI/TiO_2_ (Figure 4a) and the high imperfection amount in 20PANI/TiO_2_ (Figure 4c) supports the aggregation process (Scheme 3). Therefore, it can be concluded that 15PANI/TiO_2_ was synthesized under optimal conditions that enabled the formation of a uniform, imperfection-free PANI on the TiO_2_ particle surface, which produced both small composite sizes and a homogenous composite morphology (Figure 4b). 

### 3.6. Photocatalysis under UVA 

To validate the PANI/TiO_2_ photocatalytic properties, photocatalysis processes were performed under UVA irradiation. Wastewater was loaded with 30 mg/L of Reactive Red 45 azo dye (RR45), at pH 4 and the concentration of the catalyst was 1 g/L. It is necessary that the catalyst and pollutant establish an interaction, as the degradation process takes place on the catalyst surface. Thus, the adsorption process was performed for 30 min in the dark to obtain the adsorption/desorption equilibrium. The above approach is a common one, prior to exposing photocatalysts and dyes to UVA irradiation [11,13].

**Figure 6:**
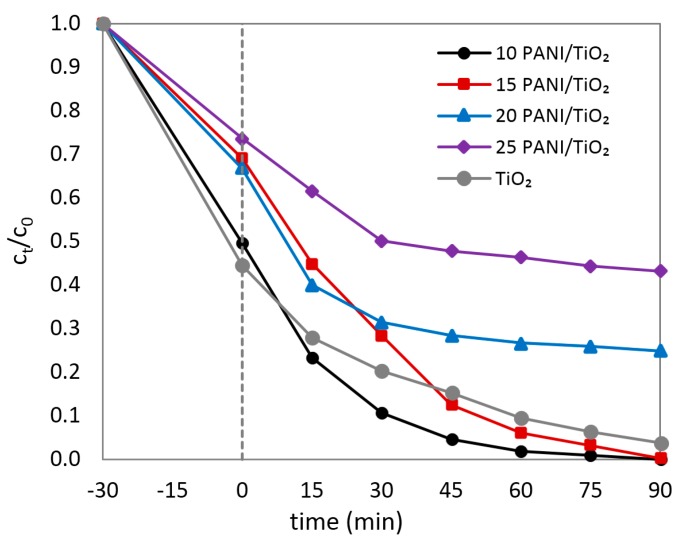
Concentration change of RR45 dye, during photocatalysis with TiO_2_ and PANI/TiO_2_ composite photocatalysts (pH = 4; γ_cat_ = 1 g/L, γ_RR45_ = 30 mg/L).

Figure 6 shows that pure TiO_2_ presented the highest ability to adsorb the dye, due to having the highest specific surface. On the other hand, the highest adsorption capacity was observed for 10PANI/TiO_2_, whilst the lowest adsorption capacity was detected for 25PANI/TiO_2_. However, the composites’ adsorption abilities are also governed by the chemical interaction of the negative anionic dyes (pH = 4) with the positively charged PANI chain [34]. Thus, differences in the composites adsorption abilities can be attributed to both the different structure of the PANI chain and to the different PANI ratio. 

However, excessive concentrations of adsorbed pollutants on the catalyst surface can be a limiting factor during photocatalysis. This particular behaviour—i.e., postponed photocatalysis—was observed for the 15PANI/TiO_2_ composite photocatalyst, as only a small portion of RR45 was removed in a period of 15 min. Following this, the photocatalysis rate was faster, as 15PANI/TiO_2_ achieved 94% of discoloration in 60 min (Figure 6). The highest discoloration rate was achieved by the 10PANI/TiO_2_ composite, with 98% in 60 min, which is an improvement in comparison to pure TiO_2_ (92%). The same trend was observed after 90 min, i.e., the 10–15PANI/TiO_2_ samples presented almost identical photocatalyst properties that were enhanced, in comparison to pure TiO_2_ photocatalyst properties.

Furthermore, the 20–25PANI/TiO_2_ photocatalytic activity significantly differed, when compared to pure TiO_2_ and 10–15PANI/TiO_2_, which can be attributed to different PANI chain structures (see Figure 2). The presence of imperfections can omit the PANI chain stretching process [35,36], which decreases PANI conductivity. In addition, due to high aniline concentrations, oxidation products with amino groups in the *ortho*-position (i.e., imperfections) [16] might be formed, which can alert the PANI chain structure and consequently polymer-dye interactions [34]. Thus, it can be assumed that the presence of the imperfections severely reduced 20–25PANI/TiO_2_ photocatalytic properties (Figure 6). On the other hand, the higher photocatalytic activity of 10–15PANI/TiO_2_ (vs. pure TiO_2_) photocatalysts, obtained when the aniline concentration was lower, is explained by the PANI-TiO_2_ synergetic effect, which is promoted when PANI is conductive and free of imperfections.

**Figure 7:**
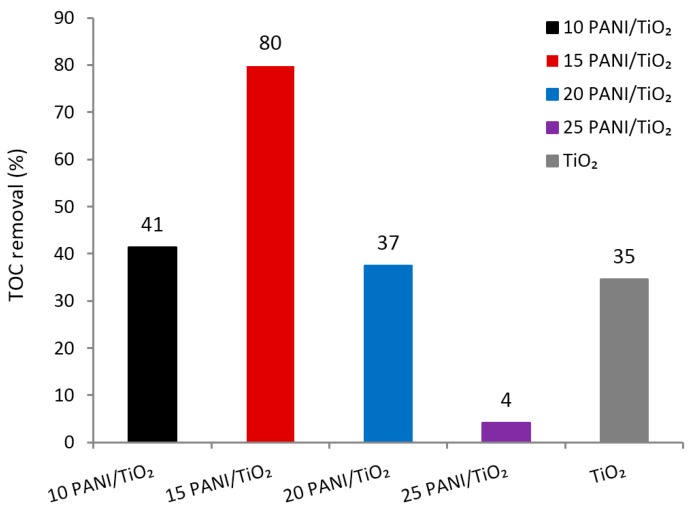
Comparison of total organic carbon (TOC) removal of Reactive Red 45 (RR45) dye after 90 min of photocatalysis (UVA) with TiO_2_ and PANI/TiO_2_ composite photocatalysts (pH = 4; γ_cat_ = 1 g/L, γ_RR45_ = 30 mg/L).

**Scheme 4:**
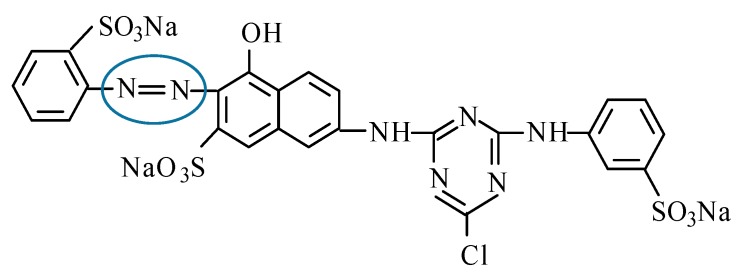
Molecular structure of Reactive Red 45 (RR45) azo dye.

The degradation of the chromophore group (–N=N–) of azo RR45 dye (Scheme 4) was determined by monitoring discoloration during photocatalysis (Figure 6), which does not necessarily indicate a degradation of complete dye molecules. Thus, to investigate the degree of mineralization of RR45 azo dye from harmless species, such as CO_2_ and H_2_O, it was necessary to determine the total organic carbon (TOC). The results are given as the percentage of removed RR45 dye, whilst the maximum dye load was taken as 100% of TOC. Furthermore, Figure 7 indicates that 80% of TOC was removed after photocatalytic degradation of RR45 using the 15PANI/TiO_2_ photocatalyst, which clearly indicates a better performance, in comparison to both TiO_2_ (35%) and 10PANI/TiO_2_ (41%). 

The degree of degradation of the pollutant molecule (RR45 dye) depends on the conditions of photocatalysis (pH, RR45 load, catalyst concentration) and on the efficiency of the catalyst itself. Figure 6 shows that the most successful discoloration was obtained with the 10PANI/TiO_2_ catalyst, though monitoring a complete degradation of RR45 indicated that 15PANI/TiO_2_ catalyst was far more effective (Figure 7). Therefore, it is obvious that the 15PANI/TiO_2_ catalyst was more efficient in removing RR45 azo dye than TiO_2_ or other composite catalysts. This means that the photocatalytic wastewater treatment by 15PANI/TiO_2_ was successful, as the catalyst was not blocked by the decomposition products (intermediates), which can be very toxic [34]. 

According to a well-known TiO_2_ photocatalyst mechanism, which absorbs photons with energy higher than 3.2 eV (Equation (1)), the excited states of electron (e_CB_^−^) and hole (h_VB_^+^) pairs are generated as follows [15]:

TiO_2_ + hν (<387 nm) → e_CB_^−^ + h_VB_^+^ (1)

where h_VB_^+^ is a powerful oxidizing agent that can mineralize dye to CO_2_ and H_2_O, according to the following equation:

h_VB_^+^ + dye → intermediates → CO_2_ + H_2_O. (2)

Equation (2) represents one of the processes that might occur after exposing TiO_2_ to UV irradiation [15]. However, photocatalytic reaction can take place only if h_VB_^+^ and e_CB_^−^ are transported to the TiO_2_ surface [6]. Since PANI is a hole acceptor, the oxidative route (Equation (2)) would be additionally promoted, especially if the well-formed and homogenous PANI is formed on the TiO_2_ surface. It was previously explained (Section 3.4) that 15PANI/TiO_2_ is covered by homogenous PANI film, and thus, its photocatalytic activity was enhanced in comparison to other composites.

Furthermore, as the degradation process is not always complete, the formed intermediates can partially block the immobile TiO_2_ active sites [13]. On the other hand, PANI active sites are mobile [37] and so they are partially immune to the intermediate blockage. Since 15PANI/TiO_2_ (vs. TiO_2_) photocatalytic performances were improved, on this basis it can be assumed that PANI in composite also protected the TiO_2_ surface from the blockage of intermediates. Therefore, PANI facilitates the photocatalyst process by enabling the PANI-TiO_2_ synergetic effect, which decreases the h_VB_^+^ and e_CB_^−^ recombination process in TiO_2_ [13]. 

### 3.7. Photocatalysis and Effect of pH Solution

pH variation can alter the surface properties of the catalyst, PANI doping, and the formation rate of both hydroxyl radicals and other reactive oxygen species, responsible for pollutant degradation. The PANI-TiO_2_ synergistic effect and TiO_2_ activity are more efficient at lower pH solutions [38]. Thus, it was of great interest to inspect the photocatalytic properties of the most efficient 15PANI/TiO_2_ catalyst at the various pH values. 

**Figure 8:**
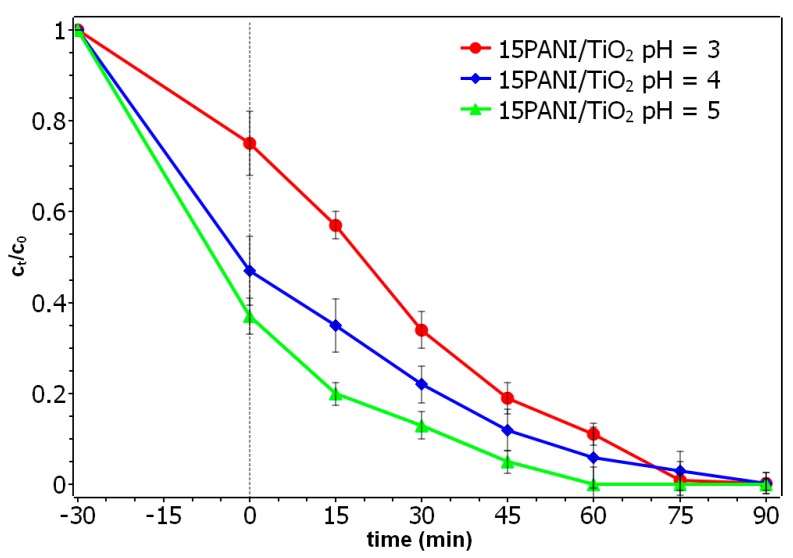
Concentration change of RR45 dye during photocatalysis (UVA) with the 15PANI/TiO_2_ composite photocatalyst at various pH values (pH = 3, 4, 5; γ_cat_ = 1 g/L, γ_RR45_ = 30 mg/L).

**Figure 9:**
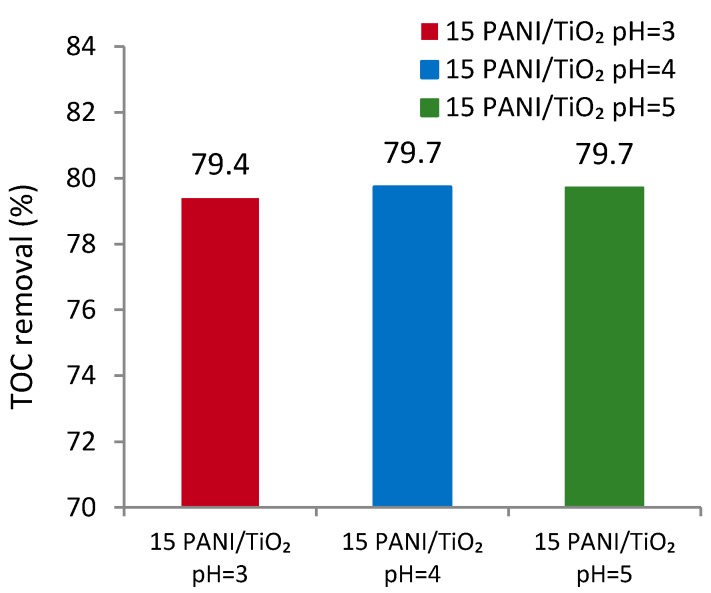
Comparison of TOC removal of RR45 dye after 90 min of photocatalysis with the 15PANI/TiO_2_ composite photocatalyst at different pH values (pH = 3, 4, 5; γ_cat_ = 1 g/L, γ_RR45_ = 30 mg/L).

Figure 8 presents the influence of pH on the photocatalytic degradation of RR45, using the 15PANI/TiO_2_ catalyst. The lowest dye adsorption (33%) was obtained at pH 3, as PANI is doped at pH = 3 [16], and thus, the HSO_4_^−^ ions prevented dye adsorption. After 90 min of UVA treatment, regardless of pH, the 15PANI/TiO_2_ photocatalyst yielded complete discoloration, as confirmed by TOC removal (Figure 9). The TOC results were almost identical, despite the pH values (≈80%), though a different RR45 degradation rate was obtained. A complete discoloration of RR45 was achieved in 60 min at pH 5, after 75 min at pH 3 and in 90 min at pH 4. Considering the obtained results and the fact that pH = 5 is more environmentally “friendly”, this value was further used in the study.

### 3.8. Photocatalysis under Solar Irradiation

**Figure 10:**
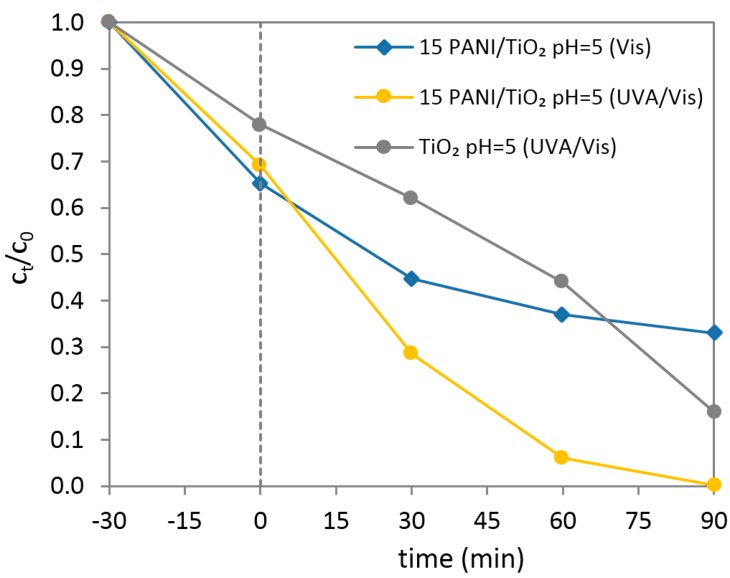
Change of RR45 dye concentration during photocatalysis with the 15PANI/TiO_2_ composite photocatalysts under UVA/Vis and Vis irradiation (pH = 5; γ_cat_ = 1 g/L, γ_RR45_ = 30 mg/L).

It is known that TiO_2_ is an excellent catalyst under UVA irradiation and a very poor one under solar (UVA/Vis) irradiation [39]. Therefore, to show that an improved 15PANI/TiO_2_ photocatalytic activity is a result of the PANI-TiO_2_ synergic effect, the RR45 degradation was conducted under UVA/Vis and Vis irradiation (Figure 10). The glass filter was used to reduce the amount of UVA irradiation. However, it is assumed that a small portion of UVA reached the composite surface.

During 90 min of UVA/Vis irradiation, the 15PANI/TiO_2_ photocatalyst completely discoloured the dye, whereas under Vis irradiation, it discoloured 77% of the dye. It is obvious that the 15PANI/TiO_2_ photocatalytic activity under UVA/Vis (Figure 10) is similar to UVA activity presented in Figure 6. However, under UVA/Vis irradiation 15PANI/TiO_2_ photocatalytic performances were superior in comparison to pure TiO_2_ that discoloured only 84% of dye after 90 min (Figure 10). The same conclusion can be obtained from TOC results under UVA/Vis irradiation, i.e., 15PANI/TiO_2_ removed 73% of TOC, whilst pure TiO_2_ removed only 27%. Therefore, it can be concluded that the PANI-TiO_2_ synergistic effect was responsible for the superb 15PANI/TiO_2_ performances.

### 3.9. Stability of Photocatalysts

**Figure 11:**
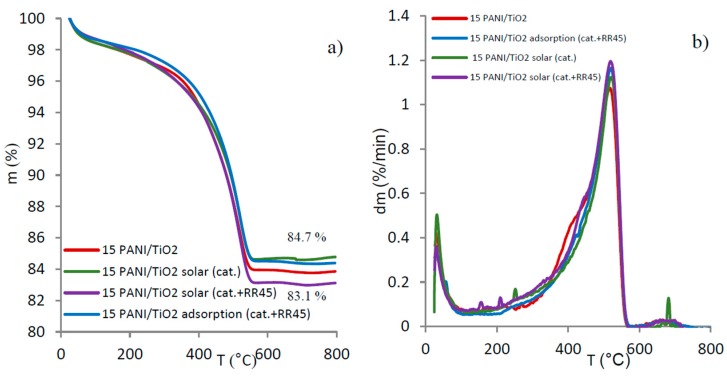
(**a**) TG and (**b**) dTG curves of 15PANI/TiO_2_ catalyst: as-prepared, after adsorption of RR45, after photocatalysis with RR45 using solar irradiation and after solar treatment without RR45.

PANI is an organic compound, so it is important to determine whether the exposure of 15PANI/TiO_2_ to solar irradiation, with or without the presence of RR45, degrades PANI within the composite. Thus, 15PANI/TiO_2_ was used in series of tests as follows: adsorption of RR45 without solar irradiation and exposure to solar irradiation with and without RR45 (pH = 5; catalyst concentration = 1 g/L, RR45 concentration = 30 mg/L). Afterwards, the catalysts used in the aforementioned tests were studied by TG (Figure 11). Additionally, the TG curve of an as-prepared 15PANI/TiO_2_ is also presented in Figure 11.

It should be noted that radicals, produced by solar irradiation [13], might induce severe PANI degradation. However, TG curves in Figure 11 reveal that the remaining char residue values varied between 83.1% and 84.7%, which indicates that the catalysts did not experience any significant degradation. Diversity in sample weight loss can be attributed to different dopant and moisture contents and to a different amount of adsorbed degradation products. The composites investigated at pH 5 (Figure 11) did not show shoulders around 400 °C (which were observed in Figure 2), since dopants were removed during photocatalytic tests. Therefore, it can be concluded that the synthesis condition for 15PANI/TiO_2_ synthesis was optimal, as the series of tests confirmed the stability of PANI in the composite.

## 4. Conclusions

PANI/TiO_2_ composite photocatalysts were successfully synthesized by the in situ chemical oxidation of aniline in the presence of TiO_2_ particles. The findings herein indicated that the aniline concentration had an observable impact on aggregation processes, morphology and photocatalytic properties. The effect of aggregation processes were enlightened while studding the sample morphology by FE-SEM and TEM images. It was concluded that the optimal aniline amount omitted the production of imperfections, and at the same time, it provided an effective barrier to TiO_2_ nanoparticle aggregation. The results indicated that the optimal aniline concentration was the one used for 15PANI/TiO_2_ synthesis (15% of aniline monomer). Since the TiO_2_ aggregation process was hindered, 15PANI/TiO_2_ resulted in the highest photocatalytic efficiency as RR45 dye degradation was taking place on the composite surface. Also, the impact of a higher aniline concentration on the polymer chain imperfections was observed, as it contributed both to the PANI chain branching and consequently, to the growth of aggregates. FT-IR spectra and conductivity measurements confirmed the presence of the conductive PANI in all prepared composites, whilst the highest conductivity was observed for the 20PANI/TiO_2_ composite. The conductivity of composites did not linearly increase with an increase in aniline concentration, as presented for the 25PANI/TiO_2_ sample. It was concluded that the conductivity of the PANI polymer reflected its chain structure, which was disrupted by imperfections, as confirmed by TG. UV-Vis results revealed the lowest reflected irradiation for 15PANI/TiO_2_, which was a result of the uniformly covered TiO_2_ surface by the equally dispersed conductive PANI.

Valorization of PANI/TiO_2_ photoactivity was conducted in wastewater treatment, where it was observed that 10PANI/TiO_2_ and 15PANI/TiO_2_ presented higher rates of discoloration than pure TiO_2_ under UVA light. TOC results point to the same conclusion, i.e., 15PANI/TiO_2_ composite demineralized 80% whereas pure TiO_2_ demineralized only 35% of RR45 dye. The higher photoactivity efficiency of the composite catalysts was explained by the achieved PANI-TiO_2_ synergistic effect. The PANI-TiO_2_ synergistic effect was additionally confirmed by UV/Vis photocatalysis, as 15PANI/TiO_2_ (vs. pure TiO_2_) yielded a more efficient catalytic process. 

In order to gain a deeper insight into the photocatalytic process of wastewater purification by 15PANI/TiO_2_ composite, it is necessary to further investigate the efficiency of water load, degradation kinetics as well as modelling of the system to achieve optimal experimental conditions. 

## Figures and Tables

**Table 1:** The components used to prepare the PANI/TiO_2_ photocatalysts.

Sample	10PANI/TiO_2_	15PANI/TiO_2_	20PANI/TiO_2_	25PANI/TiO_2_	PANI
m (PANI)/%	10	15	20	25	-
m (TiO_2_)/g	0.8	0.8	0.8	0.8	0
V (An)/mL	0.392	0.588	0.784	0.979	0.392
m (APS)/g	0.245	0.367	0.490	0.612	0.245
V (H_2_SO_4_)/mL	0.055	0.055	0.055	0.055	0.055
Final pH	2.0	2.0	2.0	2.0	2.0

PANI: polyaniline.

**Table 2:** PANI/TiO_2_ composite conductivity.

Sample	10PANI/TiO_2_	15PANI/TiO_2_	20PANI/TiO_2_	25PANI/TiO_2_
Conductivity/S cm ^−1^	2.76 × 10^−5^	3.74 × 10^−5^	6.33 × 10^−5^	1.70 × 10^−6^
